# An Effective, Versatile, and Inexpensive Device for Oxygen Uptake Measurement

**DOI:** 10.3390/jcm6060058

**Published:** 2017-06-08

**Authors:** Paule Bénit, Dominique Chrétien, Mathieu Porceddu, Constantin Yanicostas, Malgorzata Rak, Pierre Rustin

**Affiliations:** 1 INSERM UMR 1141, Hôpital Robert Debré, 75019 Paris, France; paule.benit@inserm.fr (P.B.); dominique.chretien@inserm.fr (D.C.); constantin.yanicostas@inserm.fr (C.Y.); malgorzata.rak@inserm.fr (M.R.); 2 Faculté de Médecine Denis Diderot, Université Paris Diderot—Paris 7, Site Robert Debré, 75013 Paris, France; 3 MITOLOGICS S.A.S. Hôpital Robert Debré, 48 Bd Sérurier, 75019 Paris, France; mporceddu@mitologics.com

**Keywords:** respiration assay, oxygen uptake, glycolysis, mitochondriopathy

## Abstract

In the last ten years, the use of fluorescent probes developed to measure oxygen has resulted in several marketed devices, some unreasonably expensive and with little flexibility. We have explored the use of the effective, versatile, and inexpensive Redflash technology to determine oxygen uptake by a number of different biological samples using various layouts. This technology relies on the use of an optic fiber equipped at its tip with a membrane coated with a fluorescent dye (www.pyro-science.com). This oxygen-sensitive dye uses red light excitation and lifetime detection in the near infrared. So far, the use of this technology has mostly been used to determine oxygen concentration in open spaces for environmental studies, especially in aquatic media. The oxygen uptake determined by the device can be easily assessed in small volumes of respiration medium and combined with the measurement of additional parameters, such as lactate excretion by intact cells or the membrane potential of purified mitochondria. We conclude that the performance of by this technology should make it a first choice in the context of both fundamental studies and investigations for respiratory chain deficiencies in human samples.

## 1. Introduction

A number of cell functions directly rely on the capacity of mitochondria to utilize oxygen through the mitochondrial respiratory chain (RC) [1]. Accordingly, in a number of clinical conditions, measuring the ability of mitochondria to use oxygen can shed light on the disease mechanism and/or help in establishing a diagnosis [2]. An impaired capacity for oxygen uptake is in particular observed in most primary mitochondriopathies of genetic origin. These are relatively rare disorders, but encompass numerous medical specialties [3]. In addition, both primary and secondary impairments of mitochondrial function are now regarded as instrumental in the course of a set of common diseases, including different cancers [4] and age-related neurodegenerative diseases [5]. This comes as no surprise given the role of mitochondria as a crucial turntable for the overall cell metabolism, acting as determining actor for cell differentiation, proliferation, and death. Finally, mitochondria represent a cellular sink for numerous toxins [6] to which organisms are exposed, potentially affecting their own function [7]. 

Significant defects of the RC generally result in most tissues in an elevation of the redox status of the matrix pyridine nucleotides and a reduced capacity of mitochondria to oxidize pyruvate [8]. This unused pyruvate is instead reduced to lactate by cytosolic lactate dehydrogenase and is excreted from the cells. Accordingly, the suspicion of an RC defect can be reinforced by the demonstration of abnormal acetoacetate/hydroxybutyrate (tracing the redox status of the mitochondrial pyridine nucleotide pool) and lactate/pyruvate ratios in the body fluids [2]. 

An impairment of the RC activity might also reduce mitochondrial ATP production. Under these circumstances, as to match a cellular unsatisfied demand for ATP, an activation of glycolysis, an alternative way to produce ATP (yet less efficient than the RC), will take place producing both ATP and pyruvate, thus again favoring lactate production and excretion [9]. 

**Figure 1:**
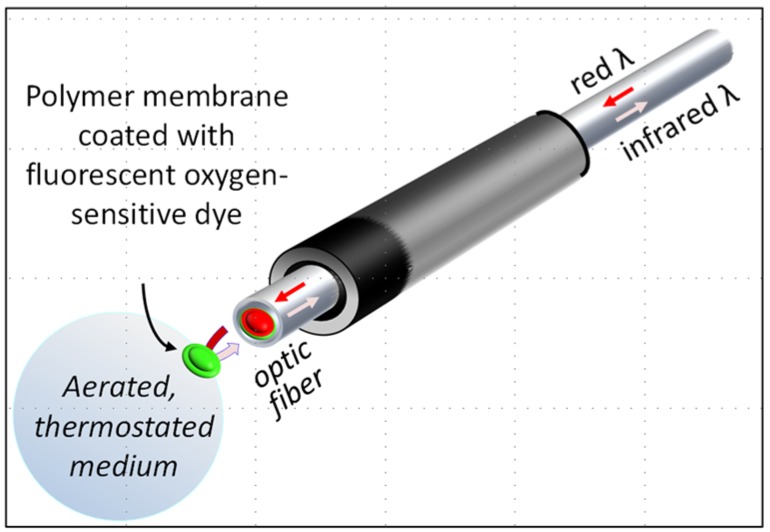
The optode device. The tip of the optic fiber (in red) is covered by a polymer membrane coated with a fluorescent oxygen-sensitive dye (in green) fixed to the optic fiber with silicone glue. The optic fiber receives red light (excitation) from, and re-emits infrared light (emission) to, an analyzer box that can be connected to a personal computer. The fluorescence of the dye is proportional to its oxygen-dependent oxidation state, which is fully reversible. Time-dependent variation of the infrared emission reflects variation of the oxygen at the membrane surface. By inserting the tip of the optic fiber into any aerated medium, it appears possible to determine at any time the oxygen tension in the medium and so to estimate oxygen consumption in the medium.

Starting with the pioneer work of Otto Heinrich Warburg during the last century [10], a number of devices have been developed to quantify the capacity of biological samples to consume oxygen. Successively using gas pressure in a closed chamber (Warburg apparatus [10]), oxygen-dependent current flow at the surface of an electrode (Clark oxygen electrode [11]), or oxygen-sensing fluorophore (oxygen extracellular fluxes; Seahorse technology [12]), methods have substantially increased in sensitivity, reducing volumes to be used from several milliliters to a few tens of microliters; however, price varied inversely, from a few to now more than €150,000. As a sensitive, versatile, and cheap alternative, we describe here the use of the Redflash technology (FireSting O2; PyroScience; Aachen, Germany) to measure oxygen uptake by various biological systems in an aqueous medium. The method measures the luminescence of an oxygen-sensitive sensor molecule covalently attached to a polymer membrane, which covers the tip of an optic fiber connected to a PC-controlled meter (Figure 1). The luminescence measurement uses red light excitation and lifetime detection in the near infrared. This represents a quite sensitive, very low-cost alternative in terms of quantifying oxygen uptake by intact cells or isolated mitochondria.

In addition, thanks to the convenient flexibility offered by the optic fiber, this device was fitted to the cuvette of a spectrophoto- or spectroflurometer, allowing for concurrent measurement of oxygen uptake plus an additional optical signal. Using such a configuration, it was possible to concomitantly and continuously measure mitochondrial substrate oxidation and membrane potential, or cell respiration and glycolysis (specifically through lactate excreted). 

## 2. Material and Methods

### 2.1. Zebra Fish Embryos

Zebrafish (*Danio rerio*) stocks of the wild-type AB strain were maintained at 28 °C in a standard zebrafish facility (Aquatic Habitat, Pentair, Minneapolis, MN, USA). Embryos were collected by natural spawning and raised under a standard 14:10 h light/dark photoperiod [13]. Developmental stages were determined as days post-fertilization (dpf), as described [14]. Three-day-old embryos were used throughout this study. 

### 2.2. Rat Liver Mitochondria

Liver mitochondria from 6-week-old Wistar Han IGS female rats (Charles River, Saint-Germain-sur-l’Arbresle, France) were isolated and purified by isopycnic density-gradient centrifugation in Percoll, as previously described [15,16,17]. 

### 2.3. Cell Culture

Primary skin fibroblasts were derived from healthy individuals and grown under standard condition at 37 °C in a 5% CO_2_, in DMEM with 4.5 g/L glucose, 4 mM glutamine as Glutamax, 10 mM pyruvate, 10% FCS, 200 μM uridine, and penicillin/streptomycin (100 U/mL). Upon confluence, cells were trypsinized, pelleted at 1500× *g*, 5 min, and used immediately for analysis.

### 2.4. Mouse Astrocytes

Astrocytes were prepared from meninges-free cerebellum of 6–7-day-old control and *Harlequin* mice with a mixed genetic background (B6CBACaAw-J/A-Pdcd8/J). The *Harlequin* mouse has been previously shown defective for complex I due to a mutation in the *Aif* gene [18]. Mice were housed with a 12 h light/dark cycle with free access to food and water. Astrocytes were plated into culture flasks in DMEM containing glucose (1 g/L) and 10% fetal calf serum at 37 °C in a 5% CO_2_. Upon confluence, flasks were shaken (180 rpm × 30 min; RockingOrbital shaker, VWR, Fontenay sous Bois, France) to remove contaminated microglia cells. Astrocytes are then detached from the culture flash by trypsin and pelleted at 1500× *g*, 5 min [19]. 

### 2.5. Ethics Statement

Details of the mouse study were approved by the Robert Debré-Bichat Ethics Committee on Animal Experimentation (http://www.bichat.inserm.fr/comite_ethique.htm; Protocol Number 2010-13/676-003) in accordance with the French and European Laws on animal protection.

### 2.6. Organism, Organs, or Cells Respiration

**Figure 2:**
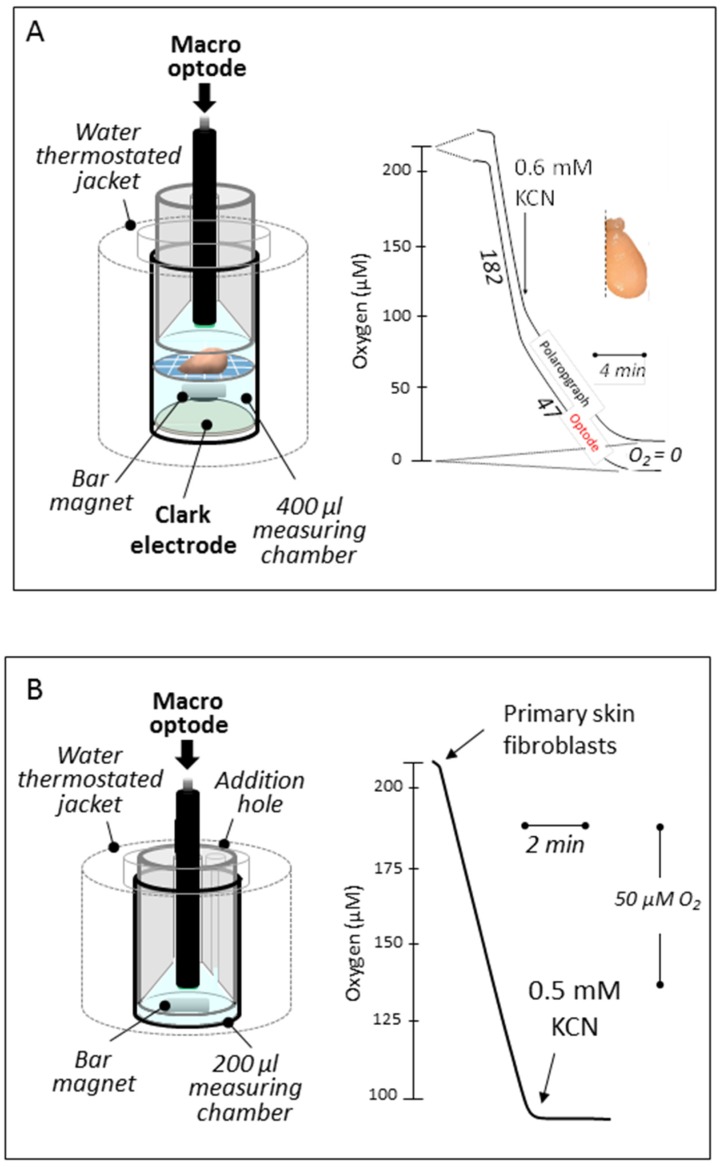
In vitro oxygen uptake by tissue sample and cells in suspension. (**A**) Both the Hansatech polarographic device (bottom) and the FireSting optode work simultaneously allowing to record strictly similar rates of oxygen uptake (about 25% resistant to 0.6 mM cyanide) by a mouse brain hemisphere place on a nylon net at mid-height in 400 μL of respiratory medium A (see Material and Methods). (**B**) Fully cyanide-sensitive human primary fibroblast respiration (about 1 × 10^6^ cells for 50 μM O_2_/min) recorded in 200 μL of respiratory medium A. Numbers along the traces are nmol/min/mg protein.

**Figure 3:**
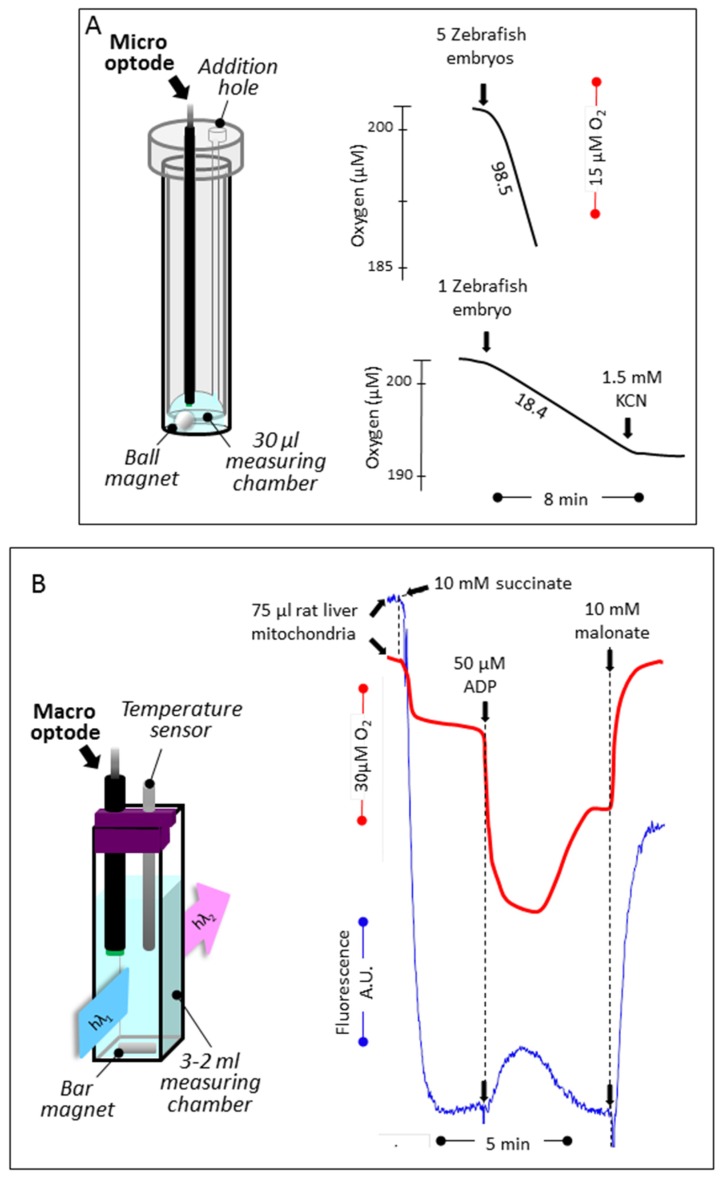
Respiration of Zebrafish embryos, and oxygen tension coupled to membrane potential determination by rat liver mitochondria. (**A**) Cyanide-sensitive respiration of Zebrafish embryos (1 and 5) measured at ambient temperature (20 °C) using a micro-optode in 30 μL of PBS (linear rates observed for 10 min). Notice the spherical magnetic stirrer avoiding to hurt the embryos. (**B**) Using an open layout, oxygen level changes linked to mitochondrial substrate oxidation was measured concomitantly to the membrane potential. Oxygen was measured using the macro-optode placed in a 3 mL quartz cell thermostated at 38 °C and magnetically stirred. Oxygen uptake (red trace) was started by the addition of succinate followed by the addition of a limiting amount of ADP (decreased level of oxygen, due to high rate of consumption; oxidation state 3 [20]), the exhaustion of which exhaustion in a higher oxygen level (oxidation state 4). The addition of malonate (a long established inhibitor of the succinate dehydrogenase [21]) fully inhibited oxygen uptake, and the level of oxygenation of the medium came back to initial value by re-equilibration with air. The membrane potential measured simultaneously (blue trace) rose upon succinate addition (quenching of rhodamine fluorescence) to drop down upon the ADP addition. After ADP exhaustion, the membrane potential rose again, while adding malonate worked to abolish most of it. Numbers along the traces are nmol/min/mg protein.

Different layouts were selected to fit the conditions imposed by the biological material selected. A first set-up (Figure 2A) allowed us to set tissue or organ samples on a nylon net (1 mm^2^ mesh) about halfway-up of the measuring chamber equipped with a handmade cap (HMC No. 1) allowing for substrate or inhibitor additions. The respiration was simultaneously measured with a macro-optode (3 mm tip diameter) inserted on the top of the chamber and with the oxygen Clark electrode at the bottom. Respiration medium A (400 μL) consisting in 0.25 mM sucrose, 10 mM KH_2_PO_4_ (pH 7.2), 5 mM MgCl_2_, 5 mM KCl, and 1 mg/mL bovine serum albumin was thermostated (37.5 °C) and magnetically stirred (high speed, stirring bar 2.0 × 5.0 mm). A second layout (Figure 2B) was used to measure oxygen uptake by cells or mitochondria with the macro-optode in a smaller volume (200 μL medium A; HMC No. 1). Using a third layout, the respiration of one to five zebrafish embryos (3 dpf) was recorded with a micro-optode (50 μm tip diameter) in a minimal volume of PBS (30 μL) (PyroScience, Aachen, Germany) (Figure 3A). The assay was carried out in a flat-bottom glass tube (6 mm diameter) positioned on a magnetic stirrer, maintained at room temperature, and equipped with a handmade cape (HMC No. 2), allowing for the micro-optode insertion and the addition of chemicals with a 5 μL syringe. A 2 mm × 2 mm ball-shaped magnetic flea slowly rotating, harmlessly aside from the embryos, was placed in the glass tube. 

### 2.7. Mitochondrial Substrate Oxidation and Membrane Potential

Oxygen uptake and membrane potential were simultaneously measured in a 37.5 °C-water-jacket-thermostated, 1.5 mL quartz-cuvette using the Flx-Xenius XC spectrofluorometer (SAFAS, Monaco, France) with a modified optical path fitted to the magnetically stirred cuvette (Figure 3B). Measurements were made using rat liver mitochondria in 750 μL of respiratory medium A with a macro-optode fitted to a hand-made open cap (HMC No. 3). Mitochondria were successively given 100 nM rhodamine, 10 mM succinate, 50 μM ADP (to ensure state 3, phosphorylating condition), and, after ADP exhaustion (state 4), 10 mM malonate, a specific inhibitor of the succinate dehydrogenase. Membrane potential variation was determined by the fluorescence change of rhodamine (503 nm λ excitation; 527 nm λ emission). 

### 2.8. Respiration and Lactate Excretion

Cell oxygen consumption and lactate excretion were measured using a similar device in 750 μL of respiratory medium A except for the handmade cap (HMC n°3), closing the cuvette yet allowing for micro-syringe (5 and 10 μL) insertion. Purified rabbit muscle lactate dehydrogenase (5 IU; EC 1.1.1.27) and 2 mM NAD^+^ were added to the cuvette to measure the lactate excreted by the cells, plus 17 mM glutamate and pig heart glutamate–pyruvate transaminase (6 IU; EC 2.6.1.2) to avoid any accumulation of pyruvate that might decrease LDH activity [22,23]. A final addition of known amounts of an NADH solution (4 μM) enabled the calibration of NADH fluorescence. Under these conditions, the rate of lactate excretion by the cell can be calculated from the rate of NAD^+^ reduction (365 nm λ excitation; 460 nm λ emission). 

### 2.9. Protein Determination and Chemicals

Protein was determined using the Bradford method [24], and all chemicals were of the highest purity grade from Sigma-Aldrich. 

### 2.10. Free 3D Printable Model Accessories

STS files corresponding to several of the layouts described in this paper (HMC No. 1, 3 and 4) are available free on demand. 

## 3. Results

### 3.1. Reducing the Volume for Oxygen Consumption 

We initially tested the macro- (extremity diameter, 3 mm) and micro-optode (extremity diameter, 50 μm) devices under the standard conditions of polarographic analysis used for more than 30 years in our laboratory to measure oxygen consumption by tissues, intact cells, or isolated mitochondria [2] (Figure 2). The macro-optode was first inserted in the top compartment of a closed, magnetically stirred, thermostated chamber equipped with an oxygen-recording Clark-electrode at the bottom compartment. The compartments were separated by a nylon grid (1 mm holes) holding a piece of tissue, yet allowing a free magnetic stirring of the 400 μL of respiratory medium. Identical responses were obtained from the optode and polarographic device (Figure 2A). With a quite similar configuration but without the electrode disk, the assay medium could be reduced to 200 μL without affecting oxygen detection by the macro-optode device (Figure 2B). Noticeably, this latter does not significantly consume oxygen (at variance with a Clark electrode). 

We next manufactured a device to use a micro-optode in a much smaller volume of respiratory medium (30–50 μL). Using a magnetically stirred (ball stirrer) 1 mL glass tube, containing 30 μL of PBS and the micro-optode device fitted to a handmade cap, it was possible to quantify the respiration of as few as 1 to 5 Zebrafish embryos, the respiration of which being proportional to the number of Zebrafish embryos studied.

### 3.2. Simultaneous Determination of Mitochondrial Oxygen Consumption and Membrane Potential

In order to simultaneously measure oxygen tension and membrane potential, we next placed the macro-optode in a magnetically stirred, thermostated quartz cell (750 μL) using an open handmade cap (Figure 3B). We then measured the changes in oxygen tension by the optode signal (red trace) and the mitochondrial membrane potential (blue trace) inversely proportional to the quenching of rhodamine-123 fluorescence (Figure 3B). 

### 3.3. Simultaneous Determination of Cell Respiration and Lactate Excretion

**Figure 4:**
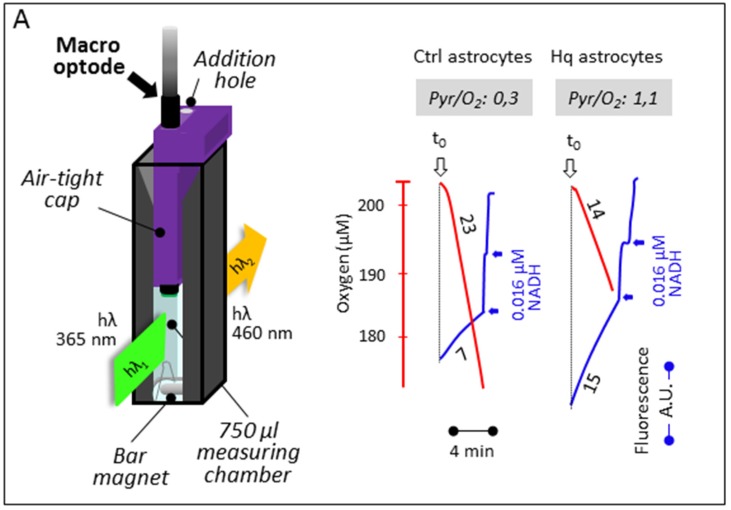

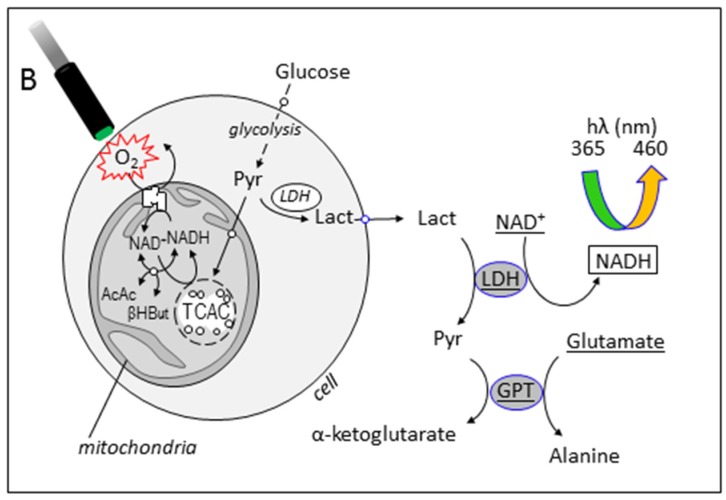
Respiration and lactate excretion by mouse-cultured astrocytes. (**A**) The macro-optode fitted to a magnetically stirred, 37.5 °C-thermostated quartz-cell by a closed cap (yet allowing for the addition of substrates and inhibitors) measures oxygen uptake due to cyanide-sensitive respiration (red traces) by control astrocytes or astrocytes prepared from the CI-defective *Harlequin* mice. The concomitant fluorometric determination of NADH (blue traces) allows for a determination of the rate of the excretion of lactate, thanks to its conversion to pyruvate brought about by added lactate dehydrogenase in the presence of added NAD^+^. Numbers along the traces are nmol/min/mg protein. (**B**) The additional presence of glutamate and glutamate transaminase avoided inhibition of the LDH reaction by accumulated pyruvate.

The macro-optode was finally inserted into a handmade cap closing a 37 °C-thermostated, magnetically stirred, 1.5 mL quartz-cell containing 750 μL of respiration medium (Figure 4A). By supplementing the medium with NAD^+^, lactate dehydrogenase (LDH), glutamate, and glutamate–pyruvate transaminase (GPT), it was possible to spectrofluorimetrically estimate the NADH accumulation due to the LDH-catalyzed oxidation of any excreted lactate to pyruvate (Figure 4B). Noticeably, in the presence of an excess of added glutamate and GPT, the pyruvate is readily transaminated to alanine and α-ketoglutarate, avoiding LDH substrate inhibition by pyruvate. To quantify the fluorescent signal, a known amount of NADH was added at the end of the assay. This allowed us in a few minutes to accurately measure the rates of oxygen consumption by respiring intact cells together with lactate production indicative of glycolytic flux. This is exemplified in the case of astrocytes prepared from control or *Harlequin* mice, the latter being defective for respiratory chain complex I [18,22].

## 4. Discussion

The comprehensive diagnostic of suspected oxidative phosphorylation (OXPHOS) defect requires the complementary assays of RC complex activity and of mitochondrial oxygen consumption [2]. Similarly, the significance of numerous base changes in the several hundred genes encoding OXPHOS components revealed by systematic sequencing can only be established by an extensive characterization of OXPHOS activities [3]. In addition to the determination of the activity of OXPHOS complexes, when possible, this includes the study of the cell respiration, the mitochondrial oxidation of various respiratory substrates, the determination of the ADP/O and respiratory control values. A complete investigation of oxidative properties supposes the use of an adaptable device allowing for the addition of multiple substrates and inhibitors in the assay medium and to register the oxygen consumption in real time. To this end, the Clark oxygen electrode that replaced the previous Warburg apparatus represented major progress, allowing for the use of much less precious material. 

Here we have shown that it is possible to use RedFlash technology to reduce (by at least two-thirds) the amount of biological sample to be studied, as compared with previous devices. This represents similar progress in terms of the biological material required and the ease of use. The device is stable for months/years, as long as the probe is kept dry and not exposed to strong light. Various optodes have been used for several years in other fields of biology [25,26,27] and a careful comparison between these devices and the Clark electrode already been reported [28]. 

In the context of screening for OXPHOS defects, an immediate benefit of using this technology is smaller muscle biopsies or blood samples needed to be taken from patients and a reduction of the amount of cultured cells to be used, i.e., fewer traumas for patients and a lower cost in terms of cell cultures. The flexibility of the optic fiber allows one to adapt the device to various specific environments, such as spectrophoto- or spectrofluorometer cuvettes. As such, it is suitable for the simultaneous determination of cell respiration and lactate cell excretion. More specific than suspending medium acidification [29], an increased rate of lactate excretion can be taken as an indication of a reduced rate of mitochondrial pyruvate oxidation or increased pyruvate production by glycolysis [22].
